# Droplet Microfluidics for the Production of Microparticles and Nanoparticles

**DOI:** 10.3390/mi8010022

**Published:** 2017-01-14

**Authors:** Jianmei Wang, Yan Li, Xueying Wang, Jianchun Wang, Hanmei Tian, Pei Zhao, Ye Tian, Yeming Gu, Liqiu Wang, Chengyang Wang

**Affiliations:** 1 School of Chemical Engineering and Technology, Tianjin University, Tianjin 300072, China; wangjm@sderi.cn; 2 Energy Research Institute, Shandong Academy of Sciences, Jinan 250014, China; liyan@sderi.cn (Y.L.); wangxy@sderi.cn (X.W.); wangjc@sderi.cn (J.W.); tianhm@sderi.cn (H.T.); zhaop@sderi.cn (P.Z.); 3 Department of Mechanical Engineering, The University of Hong Kong, Hong Kong, China; tianye@hku.hk; 4 Shandong Shengli Co., Ltd., Jinan 250101, China; perfectgu@126.com

**Keywords:** microfluidics, microparticles, nanoparticles, monodisperse, emulsion, droplet microfluidics

## Abstract

Droplet microfluidics technology is recently a highly interesting platform in material fabrication. Droplets can precisely monitor and control entire material fabrication processes and are superior to conventional bulk techniques. Droplet production is controlled by regulating the channel geometry and flow rates of each fluid. The micro-scale size of droplets results in rapid heat and mass-transfer rates. When used as templates, droplets can be used to develop reproducible and scalable microparticles with tailored sizes, shapes and morphologies, which are difficult to obtain using traditional bulk methods. This technology can revolutionize material processing and application platforms. Generally, microparticle preparation methods involve three steps: (1) the formation of micro-droplets using a microfluidics generator; (2) shaping the droplets in micro-channels; and (3) solidifying the droplets to form microparticles. This review discusses the production of microparticles produced by droplet microfluidics according to their morphological categories, which generally determine their physicochemical properties and applications.

## 1. Introduction

Monodisperse microparticles and nanoparticles with uniform sizes and morphologies are used in bio-pharmaceuticals [1,2], drug delivery applications [3,4], electro/optic devices [5,6] and catalysis [7,8,9] because of their unique properties. Many efforts are made to produce uniform microparticles with custom sizes, shapes and morphologies using traditional methods, such as precipitation polymerization [10,11], emulsion polymerization [12,13], dispersion polymerization [14,15], SPG (Shirasu Porous Glass) membrane emulsification [16,17], and layer-by-layer assemblies [18]. However, these conventional emulsion droplet methods with bulk shearing forces are uncontrollable, and the resulting droplets, especially the non-spherical particles, are disparate in size and shape. Because of the interfacial tensions between the two phases, the emulsion droplets automatically shrink into spheres, making it difficult for traditional methods to prepare quality custom shaped particles. Moreover, traditional methods are complex, inflexible and expensive. Therefore, better methods are needed to produce monodisperse microparticles with tailored sizes, shapes and morphologies. 

Droplet microfluidics techniques, including active and passive method are promising methods to generate monodisperse emulsions. The main difference between active and passive methods is based on the external forces. Active droplet generators are usually achieved by incorporating additional forces into microfluidic systems, such as electrical [19], magnetic [20], pneumatic [21], acoustic [22] and thermal [23]. Co-flow [24,25], flow-focusing [26,27,28], T-junction [29,30], step emulsification [31] and microchannel terraces [32,33] are basic passive droplet generators. The active control offers more flexibility in manipulating droplet than passive droplet generation. However, the active methods suffer from difficulties in fabrication and miniaturization. In this review, we will mainly focus on the passive methods. These approaches operate in the laminar flow region and generate one drop at a time. The conditions are identical for each droplet as it breaks off [34,35], and the produced emulsions are uniform in size, structure and composition [36]. In addition, the microfluidic devices provide much more flexibility because only the device structures need to be changed to produce complex structure droplets, such as single-emulsions, double-emulsions and multi-emulsions [37,38].

**Figure 1:**
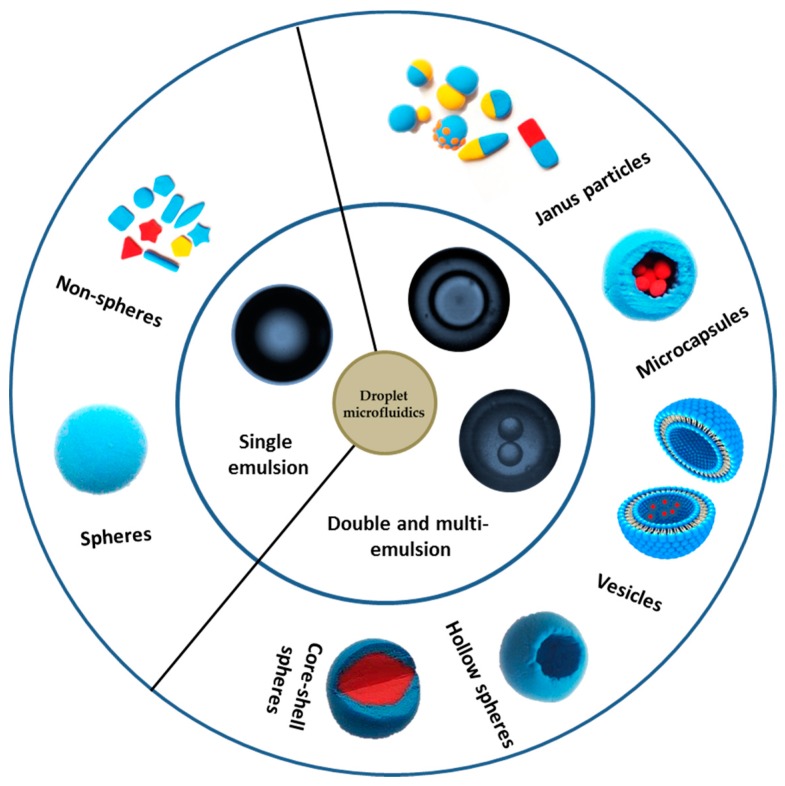
Summary of applications for microparticles and nanoparticles.

The superior properties of droplet microfluidics are advantageous for precise microparticle manufacturing, especially when used as templates to prepare microparticles and nanoparticles with various morphologies. In general, highly monodisperse emulsified droplets form in a microfluidic device and are simultaneously used as a template, and then are solidified to form microparticles and nanoparticles by chemical, photochemical or physical methods [39]. This review discusses recent advances in microparticle and nanosphere fabrication with droplet microfluidics. Because a single emulsion can be a template for solid spheres, and double or multi-emulsions can be templates for core shells, Janus or other morphology spheres, we classified the emulsion droplets according to their structures, and focused on how the emulsion droplets evolved into various structures and morphologies, as shown in Figure 1. This review provides a general background to those new to droplet microfluidics.

## 2. Microparticles and Nanoparticles with Single Emulsion Template

Single emulsions are droplets of one phase fluid dispersed in another immiscible phase fluid. The key step in forming monodisperse microparticles is to form monodisperse droplets with microfluidic devices. The most frequently used systems to generate monodisperse droplets are co-flow, cross-flow, and flow focusing, and the coefficient of variation (CV, defined as the ratio of standard deviation to the mean of the droplet radius) of droplets is usually less than 5% [40]. The size of the droplets generated from co-flow and cross-flow is often related to the dispersed channel dimension, whereas flow-focusing structure is different from the above two types. In flow-focusing, the inner fluid is hydrodynamically flow focused by the outer fluid through the orifice, and it allows generating droplets with smaller size than that of the orifice. Based on this feature, we can use a larger orifice to make droplets for those fluids with suspended particles, to minimize the probability of clogging the orifice.

Chong et al. [41] and Zhu et al. [42] gave a very detailed summary on the active and passive droplet generation methods with microfluidic devices, in which they overviewed the different droplet generators and the characteristics and mechanisms of breakup modes of droplet generation. The five breaking modes in passive generation, which are squeezing, dripping, jetting, tip-streaming and tip-multi-breaking have their own unique characteristics, and can be applied to various fields, for example, to perform chemical and biochemical reactions where droplets used as microreactors [43,44] and to synthesis microparticles with droplets as templates [45,46,47,48]. In material science, this is a superior tool for engineering micromaterials and nanomaterials, we can change the component and the structure of the droplets to produce polymer particles, inorganic nanoparticles and metal particles [49]. In this section, we will discuss how single emulsions are used as templates for generating solid particles, including spherical and non-spherical particles with different materials.

### 2.1. Spherical Particles

#### 2.1.1. Polymer Microspheres

Polymer microspheres are commonly used in pharmaceutical and medical applications. Polyvinyl alcohol (PVA), poly(lactic-co-glycolic acid) (PLGA), sodium alginate, polyethylene glycol (PEG) and gelatin microspheres have been successfully used as drug carriers [26,39,50,51,52,53,54,55,56]. Several methods, such as spray-drying, coacervation and emulsification, are used to prepare polymer microspheres. However, these conventional bulk procedures cannot be precisely controlled and result in polydispersed and irregular shapes, which limits their practical applications [57].

Xu et al. used flow-focusing geometry to generate PLGA droplets in PDMS devices [58]. They produced monodisperse particles with defined sizes ranging from 10 to 50 μm by simply tuning the flow rates of the continuous phase and the disperse phase. After loading bupivacaine (an amphiphilic drug), they found that the drug release kinetics of the monodisperse particles were different from those of the polydisperse particles produced by conventional methods. The monodisperse PLGA microparticles had significantly reduced burst releases and slower overall release rates than those of the polydisperse particles under the similar conditions. 

**Figure 2:**
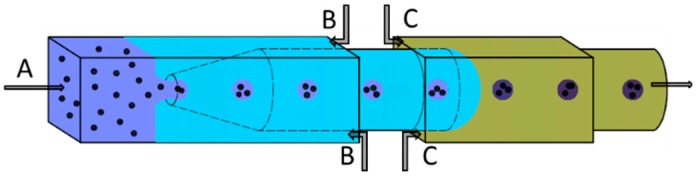
Schematic illustration of a capillary-based single emulsion device for fabricating monodisperse PNIPAm microgels. This device was used by Chu et al. [46]. **A** containing the monomer (N-isopropylacrylamide), a crosslinker (*N*,*N*’-methylene-bis-acrylamide), and a reaction initiator (ammonium persulfate) and solid polystyrene particles; **B** containing the kerosene and a surfactant; and **C** amphipathic reaction accelerator (*N*,*N*,*N’*,*N*’-tetramethyethylenediamine) dissolved in kerosene.

Chu et al. used a glass capillary-based single emulsion device as shown in Figure 2 to make monodisperse Poly(Nisopropylacrylamide) (PNIPAm) microgels [46]. The spherical voids were introduced in a controlled manner into the microgels. They found that the microgels with voids would swell and shrink in response to temperature changes faster than those with voidless microgels. In addition, the response rates were finely tuned by changing the size and number of spherical voids inside the microgels.

Seo et al. studied the emulsification of four kinds of monomer acrylates to synthesize polymer particles in a microfluidic flow-focusing device [59]. They detailed the effects of hydrodynamic conditions, channel geometry and micro-device materials on the size of the droplets. The selection of an appropriate material for the device was a vital stage in the generation of the droplets, and phase inversion occurred because of the higher affinity of the droplet phase for the material of the microfluidic device, such as glass, silicon, polydimethylsiloxane (PDMS) and polyurethane (PU). Though the channel surface could be modified by surfactants, the modified effect disappeared after several hours of emulsification. In addition, the rate of monomer polymerization with UV-light affected the quality of the sphere. Rapid polymerization caused enough heat to induce an explosion and a vacuum for low monomer-to-polymer conversion caused the sphere to collapse. Serra et al. produced polymer particles without surfactant or pretreatment in a co-flow device, and studied the effect of the viscosity of the continuous phase on the particle size [60]. They found high viscosity of the continuous phase could prevent the droplets from phase inversion and generate smaller particles for a given fluid flow rate. It could be of particular interest for the synthesis of particles with a functionalized surface.

Controlling the composition of the particles allows for a variety of functional properties. When dyes, semiconductor quantum dots, magnetic nanoparticles or liquid crystals are added to the particles, they have an optical, magnetic and actuation performance [61,62,63,64,65]. Carrying chemo-therapeutic or radio-therapeutic agents in the embolism microspheres greatly improves the treatment effects [66,67]. Magnetically guided drug carriers for medical imaging and therapeutic applications have been studied for decades and are currently ready for clinical trials [68,69]. These applications are available by adding another functional composition in the disperse phase to form stable droplet, and then by delivering the droplet into the microsphere.

#### 2.1.2. Inorganic Microspheres 

Monodisperse inorganic microspheres, including those composed of silica, carbon, and titanium, have received considerable attention for their potential applications in biomolecules, drug delivery [70,71], sensors [6] and catalysts [9]. The classical Stőber method is a general approach for the synthesis of silica spheres based on sol-gel chemistry. The synthesis involves the hydrolysis and condensation of silicon alkoxides in alcohol solvents with ammonia as the catalyst, and produces monodisperse silica spheres of predetermined sizes in the range 0.05–2 μm [72]. However, the particle sizes are not precisely reproducible with this method. Liu et al. extended this method to prepare monodisperse resorcinol-formaldehyde resin polymers (RFs) and carbon spheres, and the particle sizes of the RFs and carbon spheres were tuned from 200 nm to 1000 nm by varying the concentration of the reactant [73]. However, this method is not generally used to prepare other materials, especially when preparations require precise control over the particle sizes across wider ranges and shapes. 

**Figure 3:**
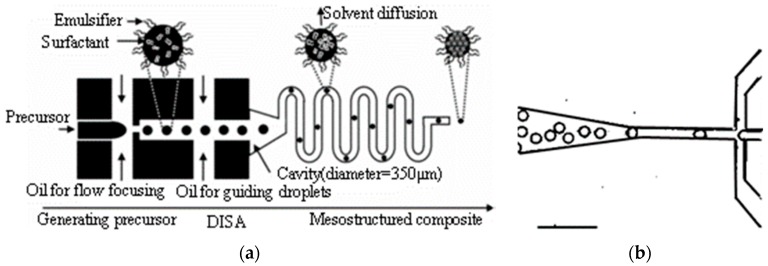
(**a**) Schematic illustration of the synthesis of OMS particles using microfluidic DISA used by Lee et al. [47]. Reproduced with permission from Lee, I., et al., *Advanced Functional Materials*; published by John Wiley and Sons, 2008. (**b**) Optical microscopy image of droplets of the silica precursor solution emulsified in a flow focusing microfluidic device in hexadecane from Carroll et al. [74]. The channel dimensions of the orifice are 25 μm in width and 30 μm in length. The scale bar is 100 μm. Reproduced with permission from Carrol, N.J., et al., *Langmuir*; published by American Chemical Society, 2008.

Lee et al. [47], Carroll and another researchers [74,75] reported a one-step method to manipulate ordered mesoporous silica (OMS) in a microfluidic device. This method combined a microfluidic emulsification technique and a rapid solvent diffusion induced self-assembly (DISA) technique. Monodisperse droplets were generated at the flow-focusing orifice and assembled into mesostructured silica/surfactant composite spheres within the microchannel, as shown in Figure 3. The sizes and the surface morphologies were easily controlled by changing the synthesis parameters, such as the geometry of the microfluidic channels, the flow rate of the precursor solution and oil, and the type of oil.

#### 2.1.3. Noble Metal Nanospheres

Noble metal nanoparticles, such as gold, silver and platinum, are interesting materials because of their size and shape dependent properties [49,76,77,78,79,80,81,82,83,84]. However, the individual nanoparticles tend to coagulate and precipitate to lower the surface free energy. Therefore, these materials are difficult to use to obtain a desired size and size distribution. 

The control of the crystal structure of the nanoparticles is another key issue in nanoparticle synthesis. Heat and mass control is very important to crystal growth, and the droplet microfluidics device provides a unique platform to precisely control heat and mass, which results from the fast heat and mass-transfer rates in the microchannel due to the short diffusion pathways induced by the small characteristic lengths of microfluidic device.

Gold nanoparticles have received considerable attention because of their broad range of applications. Recently, gold nanoparticles were synthesized from spherical gold nanoparticle seeds <4 nm in size in the microfluidic device [85]. Various shapes such as spheres, spheroids, rods and extended sharp-edged structures were obtained by tuning the concentrations of reagents and feed rates of the individual aqueous streams.

#### 2.1.4. Semiconductor Nanospheres

Droplet microfluidics are commonly used in synthesizing nanoparticles at room temperature [86], but the pyrolytic synthesis of high quality semiconductor nanocrystals, such as CdSe, requires higher reaction temperatures of 200–350 °C, which renders this method impractical. The droplets and carrier fluids should be stable, non-interacting, non-volatile and immiscible from ambient to reaction temperatures, and the microfluidic reactor must have thermal and chemical stability [87]. 

**Figure 4:**
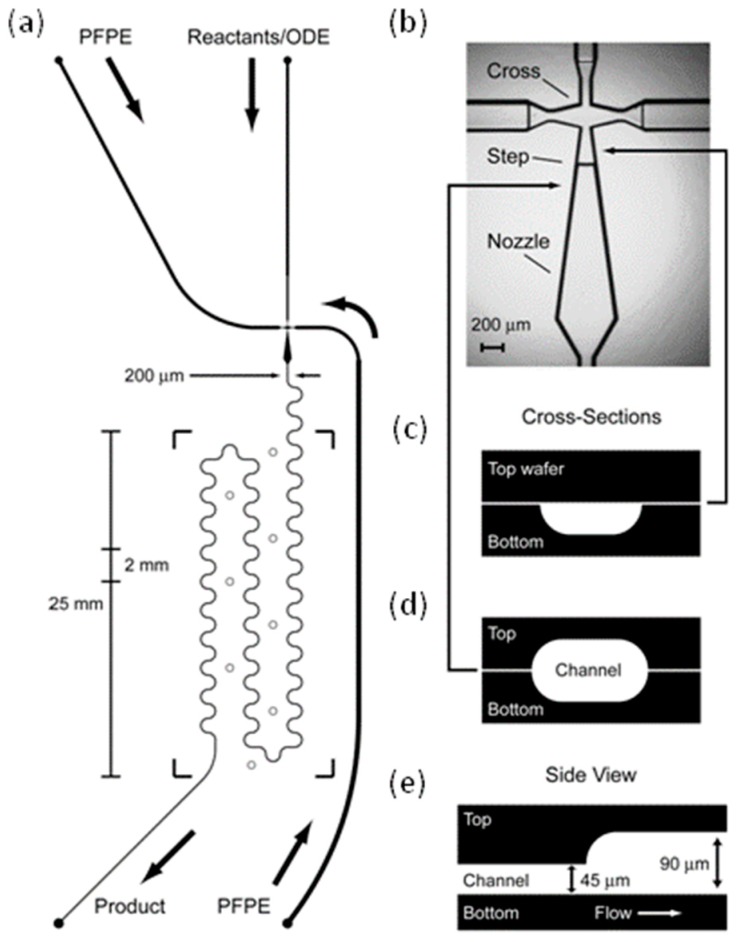
The microreactor channel design with droplet jet injector used by Chan et al. [87]: (**a**) Channel schematic showing dimensions, inlets (●), thermocouple wells (○), and boundaries of Kapton heater [87] (**b**) Optical micrograph of droplet injection cross. ODE is injected into the top channel, while the PFPE is injected in the side channels (**c**) Lateral “D”-shaped cross section of channel etched on the bottom wafer only (**d**) Cross-section of ellipsoidal channel etched on both top and bottom wafers. (**e**) Axial cross-section showing the 45 μm stepped up in channel height. Reproduced with permission from Chan, E.M., et al., *Journal of the American Chemical Society*; published by American Chemical Society, 2005.

Chan et al. used octadecene (ODE) as the solvent, long-chained perfluorinated polyethers (PFPEs) with high-boiling points as the continuous fluids and glass as the microreactor material. All fluids and device materials were stable at the reaction temperature [87]. However, this system had a low interfacial tension (γ) (5–25 mN/m) and a high viscosity (μ) (>100 mP·s) for the high-boiling PFPEs, which induced a high value of *C*_a_ (*C*_a_ = μν/γ) and a low value of viscosity ratios (λ where λ = μ_disperse_/μ_continuous_). This was undesirable for droplet formation because the interfacial velocity (γ/ν) was not fast enough relative to ν (m·s^−1^) to relax the strained interface into forming droplets, in addition, it need large values of shear rate to rupture the interface at low viscosity ratios, which can generate high pressures with viscous PFPEs as carrier fluids [27,88,89].To solve this problem, Chan et al. designed a microdevice with a stepped microstructure increasing in channel height, as shown in Figure 4. With this device, ODE droplets in Fomblin Y 06/6 PFPEs were generated at a flow-focusing orifice, and CdSe nanocrystals were produced when the droplets going through the glass microreactor had temperatures of 240–300 °C, although *C*_a_ was 0.81 and λ was 0.035.

### 2.2. Non-Spherical Particles 

Non-spherical particles offer unique properties compared to those of spherical particles [90,91,92,93]. For example, in optics, rod-shaped particles often have superior optical properties due to the optical antenna effect. Prior studies suggested that anisotropically shaped nanoparticles can avoid bio-elimination more effectively than spherical particles under the same conditions [94]. These findings are promising and will lead to additional studies on irregular shapes and corresponding applications [49,85,95,96,97,98]. Many strategies were developed to fabricate non-spherical particles, including template molding [99], seeded emulsion polymerization [100] and self-assembly [101]. However, these methods are still difficult for producing high quality, monodisperse, non-spherical particles with tailored geometries and shapes.

Recent advances in droplet microfluidic technologies offer new approaches for the fabrication of non-spherical particles. One approach is to confine the droplets in microfluidic channels with different sizes and shapes. If the volume of the droplet is larger than that of the largest sphere which could be accommodated in the channel, the droplet will be deformed into a disk, ellipsoid or a rod, and the non-spherical particles can be generated after they are solidified in the confined channel [49,102]. Another approach is through the combination of photo-chemistry and photomasking; the photomask with desired patterns is used as a template for the final particles. When the droplet periodically flows through the mask, photo-initiated polymerization will occur. With this approach, polymer particles with complicated shapes can be generated [91,103].

**Figure 5:**
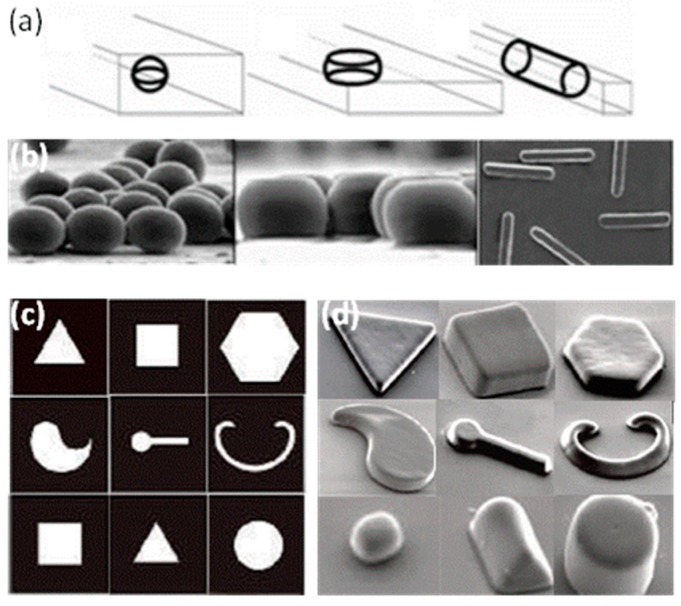
Non-spherical particles formed using the two approaches. (**a**) The spherical droplets deformed by the confinement of the outlet channel; (**b**) The optical microscopy images of particles: ellipsoids, disks, rods with (**a**) approaches [49]. Reproduced with permission from Xu, S., et al., *Angewandte Chemie*; published by John Wiley and Sons, 2005; (**c**) The transparency mask used to make nonspherical particles; (**d**) The SEM images of corresponding particles with (**c**) approaches [103]. Reproduced with permission from Dendukuri, D., et al., *Nature Materials*; published by Nature Publishing Group, 2006.

Xu et al. produced particles with different sizes and shapes using the first method [49]. They produced monodisperse droplets in a flow-focusing device, shaped the droplets in the confined channel, and solidified these droplets in situ. This method is applicable to a variety of materials, such as gels, metals and polymers. Some products are shown in Figure 5a,b. Dendukuri et al. easily produced complex and multifunctional particles using the second approach [103]. They synthesized various shapes, such as polygonal shapes, non-symmetric or curved objects, and high-aspect-ratio objects, as shown in Figure 5c,d. The shape and the size of particle is only controlled by the mask, and the morphology and the chemistry of the particles can be independently chosen to form a large number of unique particles for applications in coding, drug delivery and biosensors.

## 3. Microparticles with Double or Multi-Emulsions as the Template

**Figure 6:**
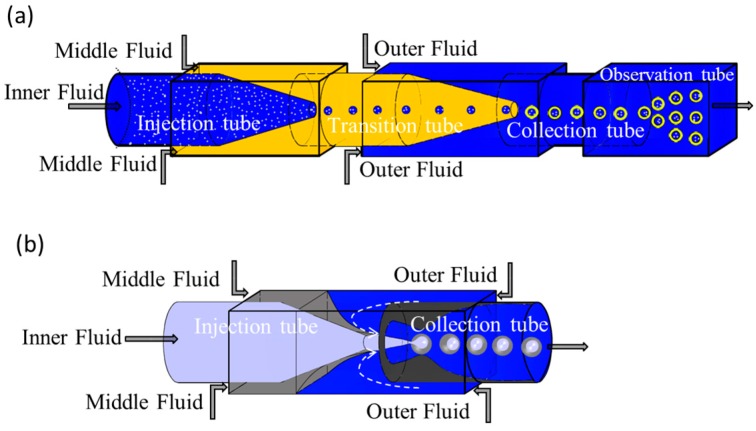
Fabricate of double emulsions in microfluidic devices. (**a**) Schematic of a capillary microfluidic device that combines double co-flowing geometry adapted from Figure 1 in [107]; (**b**) Schematic of a capillary microfluidic device that combines co-flow and flow-focusing geometry adapted from Figure 1 in [37].

Double or multi-emulsions are droplets with smaller droplets encapsulated in larger drops. Core shell microparticles are typically made using double emulsion droplets as templates. Theoretically, micro-devices combined with co-flowing and/or flow-focusing geometries can easily produce monodisperse double or multiple droplets, most of which are capillary microfluidic devices, as shown in Figure 6. There are three fluids flowing in different capillaries, including the inner fluid in the injection capillary, the middle and the outer fluid in square capillary. The inner fluid is sheared by the middle fluid to form single droplets, and the middle fluid containing one or more single droplets is pinched off by the outer fluid to form double or multiple droplets. This technique eliminates the difficulties of precisely controlling the shell thickness, secondary nucleation and aggregation, and non-uniform in the traditional processes [104,105,106]. However, the precise size and morphology control in this technology is very important and still a challenge to the preparation of microparticles.

### 3.1. Size Control of Core Shell Microparticles

**Figure 7:**
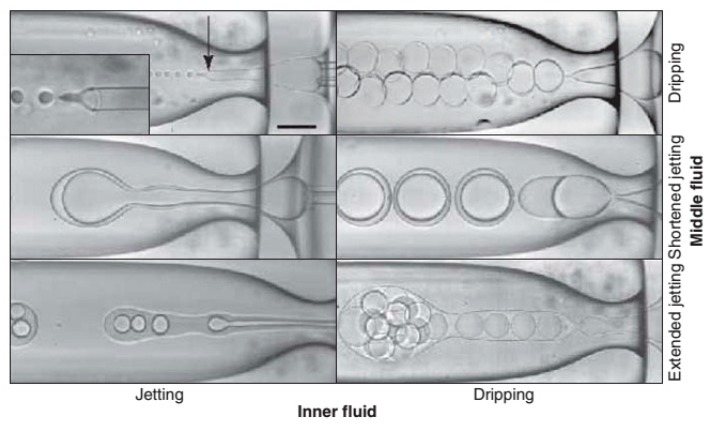
Different morphologies of double emulsions produced by a microcapillary device [109]. Reproduced with permission from Utada, A.S., et al., *MRS Bulletin*; published by Cambridge University Press, 2007.

Accurate control of the particle size is essential and in turn affects the release of inner active materials [108]. Dripping and jetting are two droplet formation regimes for each inner and middle fluid, as shown in Figure 7 [109]. The dripping regime may be the best choice to form a controllable core shell structure. The inner and outer drops should all be formed in the dripping regime to produce uniform microparticles, and the thickness of the shell can be precisely controlled. 

**Figure 8:**
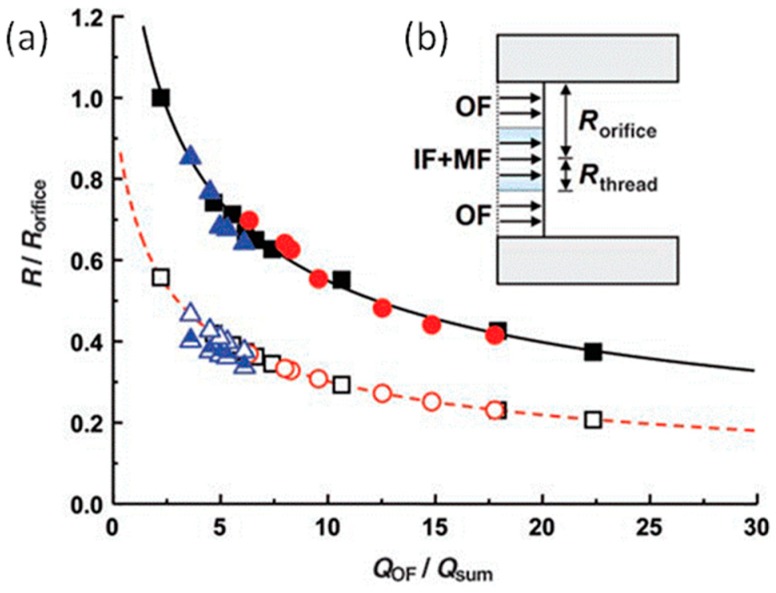
(**a**) Dependence of *R*_thread_*/R*_orifice_ on the scaled flow rate *Q*_OF_*/Q*_sum_ [110]. The open symbols represent the *R*_thread_ for different liquids and double emulsions consisting of a single silicon drop surrounded by a liquid shell (3 *Q*_IF_ = *Q*_MF_, triangle). The dashed line represents the predicted *R*_thread_. *R*_drop_ values are represented with solid identical symbols. Half-filled triangles correspond to the radius of the internal droplets of the double emulsions. The solid line represents the predicted *R*_drop_; (**b**) The flat velocity profile of the flow as it enters the capillary tube [110]. Reproduced with permission from Kim, J.W., et al., *Angewandte Chemie*; published by John Wiley and Sons, 2007.

Kim et al. used a microfluidic device to illustrate the predicted model of the radius droplet, as shown in Figure 8b [110]. At lower flow speeds, droplets will form in the dripping regime where droplets are very close to the orifice, and the mass flux is related to cross-sectional area. The size is controlled by the ratio of the flow rates of the sum of inner and middle fluids to the outer fluid (*Q*_sum_*/Q*_OF_). Equation (1) gives the relationship of the flow rate (*Q*) and each radius (*R*), where *R*_thread_ is the radius of the fluid thread that breaks into drops, and *R*_orifice_ is the radius of the exit orifice. The values of *R*_thread_/*R*_orifice_ are predicted from Equation (1), with no adjustable parameters, and are consistent with the measured values in Figure 8a (open symbols). A comparison of the measured radii of the drops and the thread shows that *R*_drop_ = 1.82*R*_thread_ [111]. Based on the above research, *R*_drop_ can be predicted, which provides important guidance in creating double emulsions of a desired size.

(1) $$\frac{Q_{\mathrm{sum}}}{Q_{\mathrm{OF}}}=\frac{\pi R_{\mathrm{thread}}^{2}}{\pi R_{\mathrm{orifice}}^{2}-\pi R_{\mathrm{thread}}^{2}}$$

Chang et al. designed a two co-axial capillaries microfluidic device to produce double droplets, from experiments they extracted an empirical law to predict core and shell sizes [112]. Since the core and the overall core shell drop has the same formation time, the core and the shell size could be predicted using the following equations, shown as Equations (2) and (3) (*Q*_I_ and *Q*_M_ are the inner and middle fluid flow rate respectively):

(2) $$d_{\mathrm{core}}=\sqrt[{3}]{\frac{Q_{I}}{Q_{I}+Q_{M}}}d_{\mathrm{drop}}$$

(3) $$d_{\mathrm{shell}}=\frac{1}{2}(1-\sqrt[{3}]{\frac{Q_{I}}{Q_{I}+Q_{M}}})d_{\mathrm{drop}}$$

### 3.2. Morphology Control of Core Shell Microparticles

**Figure 9:**
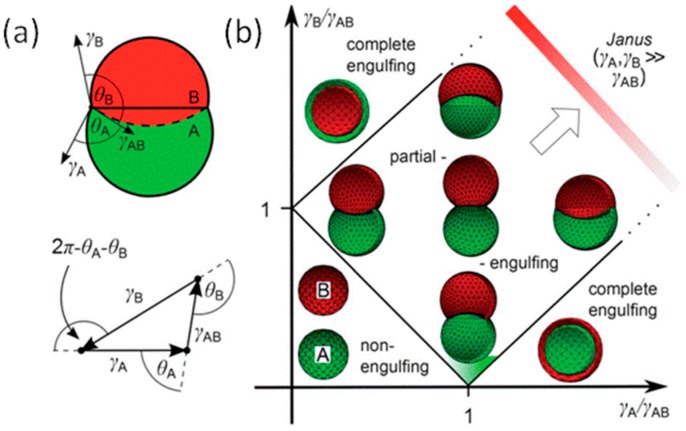
(**a**) Schematic of a double droplet with indicated contact angles θ_A_ and θ_B_ and the Neumann’s triangle [115]; (**b**) Stability diagram representing the possible morphologies of a double droplet of phase A and B in the case κ = V_B_/V_A_ = 1, κ is the ratio of the liquid volumes [115]. Reproduced with permission from Guzowski, J., et al., *Soft Matter*; published by Royal Society of Chemistry, 2012.

As found by Dowding and Shum, when the polymer was formed by phase separation, the emulsion stabilizer had to be adjusted to control the particle morphology [113,114]. For combinations of a range of liquids, the final equilibrium morphology can be a core shell, or “acorn”-shaped, as shown in Figure 9. The transitions between different topologies of the double droplet can be described as the interfacial tensions model [115]. In the interfacial tension model, the balance of forces acting on the three-phase contact line is expressed in the form of relations (Neumann triangle Law, Figure 9a) between the contact angles and the interfacial tensions:

(4) $$\gamma_{\mathrm{AB}}{\cos\theta }_{B}+\gamma_{B}+\gamma_{A}(\theta_{A}+\theta_{B})=0$$

(5) $$\gamma_{\mathrm{AB}}{\cos\theta }_{A}+\gamma_{A}+\gamma_{B}(\theta_{A}+\theta_{B})=0$$

The existence and type of solution of Equations (4) and (5) depends on the values of the interfacial tensions. Guzowski and Korczyk used interfacial tensions to describe the transitions between different topologies of the droplets, marked by solid lines in Figure 9b [115]. They found three possible equilibrium topologies:

1. Complete-wetting: γ_B_ > γ_AB_ + γ_A_, where a droplet of phase A is entirely encapsulated by phase B; vice versa (γ_A_ > γ_AB_ + γ_B_), typical core shell structure;
2. Non-wetting: (γ_AB_ > γ_A_ + γ_A_), droplets of phase A and B are separated by the outer phase;
3. Partial-wetting: droplets of phase A and B have a common interface and are both exposed to the external phase, corresponding to acorn-shaped or Janus.

This model has been generally applied to rationalize the particle morphologies observed when the polymer was caused to phase separate within the emulsified droplets, and in the presence of various core oils and aqueous emulsifier combinations [116]. By controlling the composition of the organic middle phase, the evolvement process will be conveniently transformed from initial core shell to the desired acorn-like configuration. Each of these morphologies has different potential applications. For example, the complete wetting state can be used for the encapsulation of active compounds, and partial wetting can be used to synthesize asymmetric and non-spherical functional particles [117].

### 3.3. Janus Particles

**Figure 10:**
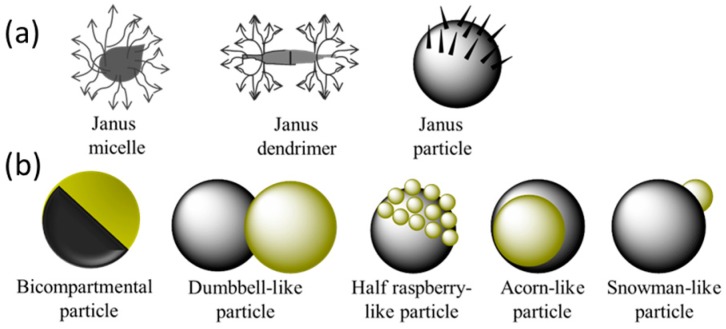
Schematic representation of Janus-like morphologies. (**a**) Extended Janus morphologies; (**b**) Solid Janus particles. (Note: spheres symbolize particles, diamonds and triangles symbolize chemical functions).

Janus particles are a class of anisotropic colloids, two sides of which have different compositions, polarities or surface modifications [117,118]. These particles have a wide range of potential applications in emulsion stabilization and dual-functionalized optical, electronic, sensor devices and incompatible drug delivery [119,120,121,122,123]. The “Janus” term has been also used for describing asymmetric dendritic macromolecules or unimolecular micelles based on block copolymers in solutions. Nevertheless, we will focus on hard and permanent Janus structures in this review, including biocompartmental, dumbbell-like, snowman-like, acorn-like and half-raspberry-like particles, as shown as Figure 10. In terms of materials, the reported Janus includes hydrogels and amphiphilic Janus. 

#### 3.3.1. Hydrogel Janus

Hydrogel Janus is composed of two hydrogel phases produced from two completely immiscible hydrophilic monomer fluids. Unlike the principle for fabricating homogeneous particles, here, two separate streams are co-flowing through the same channel of the microfluidic device. However, the two fluids must remain parallel and the interface between them must be stable at different temporal and spatial scales, and any perturbations can lead to the formation of particles with mixed internal morphologies instead of particles with two distinct sides [24].

**Figure 11:**
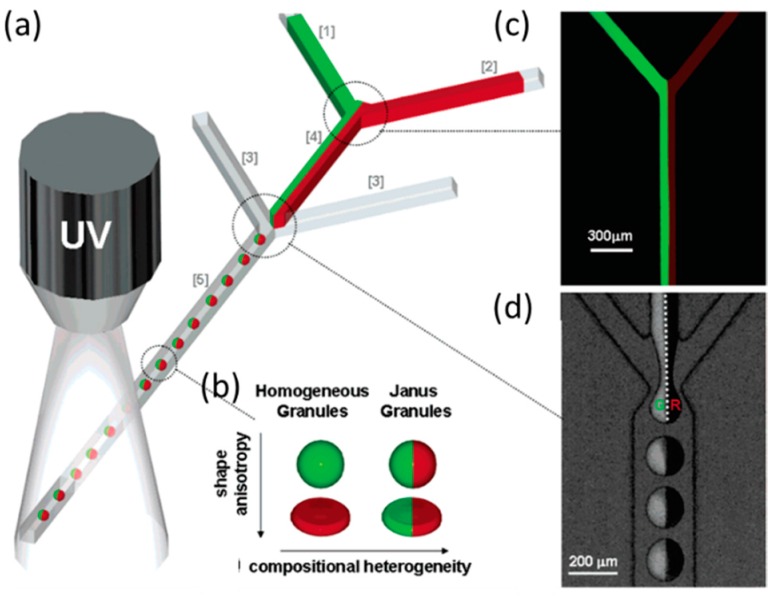
(**a**) Schematic representation of sheath-flow microfluidic device used to produce monodisperse colloid-filled hydrogel granules; (**b**) Schematic view of granule shapes and compositions explored; (**c**) Fluorescent image of Y-junction formed by inlets [1,2] for the production of Janus spheres; (**d**) Backlit fluorescence image (green excitation) illustration that the fluorescein isothiocyanate (FITC)-silica microspheres remain sequestered in the left hemisphere of each granule generated. Pictures are from [124]. Reproduced with permission from Shepherd, R.F., et al., *Langmuir*; published by American Chemical Society, 2006.

**Figure 12:**
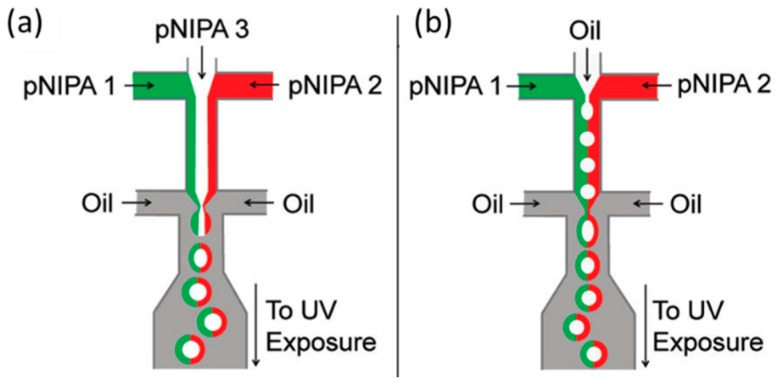
Formation of Janus microgels and microshells [125]. (**a**) Schematic of a microfluidic device forming aqueous droplets from three independent semidilute pNIPPAm solutions. The center phase (white) is assembled in the core of the droplets, whereas the right- and left-flowing phases (red and green) form the shell (**b**) operating the device in a modified way yields oil-water-oil double emulsions with Janus-shaped middle phases. Reproduced with permission from Seiffert, S., et al., *Langmuir*; published by American Chemical Society, 2010.

Shepherd and Conrad reported a scalable microfluidic assembly route for creating monodisperse silica colloid-filled hydrogel Janus [124]. Drops were formed by shearing a concentrated silica colloid-acrylamide aqueous suspension in a continuous oil phase using a sheath-flow device, as shown in Figure 11. Then, they immobilized the colloids within each drop by photo polymerizing the acrylamide to form a hydrogel. Seiffert et al. demonstrated a microfluidic technique to produce functional Janus microgels from prefabricated, cross-linkable precursor polymers [125]. This approach separated the particle formation from the synthesis of the polymer material, which allowed the droplet templating and functionalization of the matrix polymer to be controlled independently. Therefore, Janus particles were created with very specific, well-defined modifications of the two sides, even on a molecular level. In addition, they fabricated hollow microcapsules with two different sides (Janus shells), using the method as shown in Figure 12, and the size of the resultant droplets were controlled by adjusting the fluid flow rates and channel geometry.

Although the mixing between liquids in a laminar flow was weak, it was enhanced by the hydrodynamic focusing of liquid threads before the break-up into droplets, which led to a gradual change in composition along the direction normal to the interface [126]. This limits the Janus application since a sharp interface between the phases is required.

#### 3.3.2. Amphiphilic Janus

Amphiphilic Janus is formed from different immiscible fluids using double-emulsion droplets as templates. This method eliminates the problem of the mixing in the interface, however, the interfacial tension γ or spreading coefficients *S_i_* must be within a certain range to assure the stability of double droplets within the partial-wetting area (in Figure 9). Thus, the portfolio of chemicals from a broad range of immiscible fluids is a key problem in the preparation of Janus particles. 

Chen et al. fabricated acorn-like particles using W/O/W double emulsions as templates in PDMS microfluidic devices [80]. Moreover, the inner and the outer droplet size were adjusted by varying each fluid flow rate, and the swelling of the particles was controlled by varying the cross-linker concentration. Dendukuri et al. reported the synthesis and self-assembly of wedge-shaped particles bearing segregated hydrophilic and hydrophobic sections using continuous flow lithography technology (CFL) [127]. Monodisperse dumbbell-like hybrid Janus microspheres with organic and inorganic parts were prepared using fused perfluoroplyethers (the organic phase) and hydrolytic allylhydridopolycarbosilane (the inorganic phase) droplets as the template in a cross-flowing microfluidic device [128]. The particles had distinctive surface properties. The hydrophobic hemisphere had a smooth surface and the hydrophilic region had a rough, porous surface.

### 3.4. Microcapsules

Microcapsules are commonly used in pharmaceuticals, foods, cosmetics and absorbent [85,129,130,131]. The solid polymer shells provide effective encapsulation, protect the encapsulated drug or the active materials from hazardous environmental conditions, and give a release profile for a desired period. Though solid microspheres can also be used as drug delivery supporters, the drug distribution is largely dependent on the microsphere size [132,133], and the active-release mechanism is the diffusion-degradation [57], which limits improvement for medication compliance. 

Microcapsules provide another way to control the drug-release rate and the release mechanism [2,90]. First, when a drug is localized in the core matrices, the shell prolongs the diffusion path of water-in and drug-out, and hence lowers the initial burst release [134]. Second, altering the properties of the shell, such as the shell thickness, may change the active transport kinetics [90]. For example, when increasing the polylactic acid (PLA) shell thickness to 10μm, the release profile will shift from a biphasic shape for pure PLGA microspheres to a zero-order piroxicam release [135]. However, it is still difficult to produce a microcapsule with a predicted size and morphology. 

**Figure 13:**
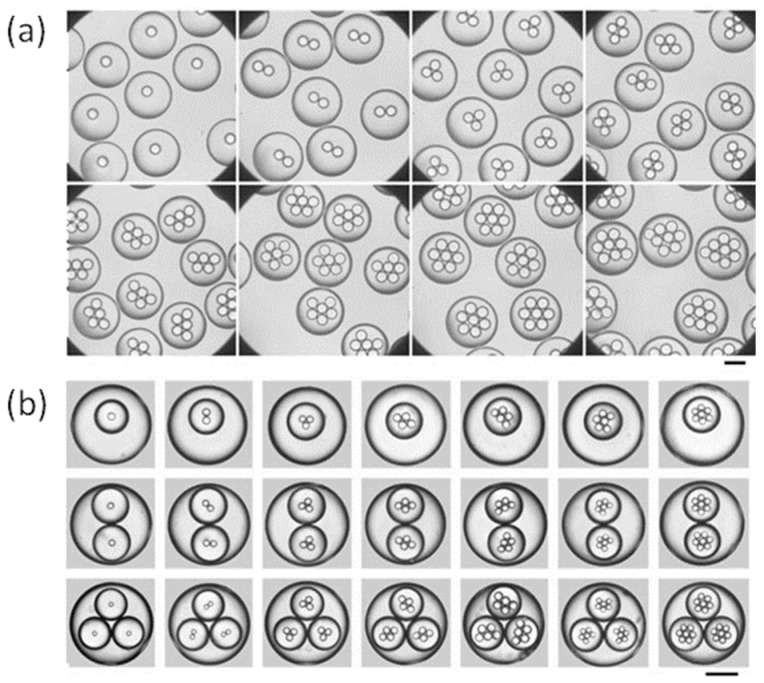
(**a**) Optical micrographs of double emulsions that contain a controlled number of inner droplets; (**b**) Optical micrographs of triple emulsions that contain a controlled number of inner and middle droplets. The scale bar in all images is 200 μm. Pictures are from [136]. Reproduced with permission from Chu, L.-Y., et al., *Angewandte Chemie International Edition*; published by John Wiley and Sons, 2007.

Equations (1)–(3) show a quantitative analysis of the relationship between the flow rate and the diameters of the inner and outer drops, and predicts the number of inner droplets [62,112]. Chu and Utada fabricated highly monodisperse multiple emulsions with controlled sizes and inner structures using capillary microfluidics, and obtained a liner relationship between the relative flow rate with drop diameters for both the inner and outer drops, and with the number of encapsulated droplets in the double emulsions [136]. By simply incorporating alternate emulsification schemes, more complicated multiple emulsions and microcapsules can be fabricated, as shown in Figure 13.

Carbon microspheres are of great interest due to their potential applications as cellular delivery vehicles, drug delivery carriers and absorbents [137,138,139,140]. Zhang et al. prepared monodisperse poly(furfuryl alcohol) (PFA) hollow microspheres by the microfluidic technique in a T-junction device through interfacial polymerization of FA in H_2_SO_4_ solution droplets. After pyrolysis, they produced carbon microspheres with mean particle sizes of 0.7–1.2 μm [141,142]. If some solid nanobeads or microbeads are added in the inner fluid, this method can be adapted to make microcapsules with controlled holes [107]. Other functional nanoparticles such as SiO_2_, Au, Co, Fe_3_O_4_ and fluorescence nanoparticles have been introduced into the inner fluid to fabricate functional microcapsules [117,143,144].

### 3.5. Vesicles

An ideal encapsulating structure should not only have high encapsulation efficiency, but also should be easily triggered to release the actives. Vesicles are a good choice for an encapsulating structure. Vesicles are a compartment of one fluid enclosed by a bilayer of amphiphilic molecules, such as phospholipids, polypeptides and diblock copolymers, which correspond to liposomes and polymersomes. The bilayer membrane can encapsulate hydrophilic and hydrophobic molecules simultaneously. Thus multiple drugs with different properties stored in a single carrier can be released at the same time. The bilayer membrane also induces semi-permeability of small molecules, such as water, leading to inflated or deflated responses to osmotic pressure differences between the aqueous core and surrounding environment.

Liposomes are biocompatible vesicles with phospholipid bilayers. They have attracted much attention because phospholipids constitute the majority of biological membranes found in nature, such as plasma membranes. Thus, they have great potential for encapsulation and targeted drug delivery considering their biocompatible properties. The disadvantage is that they are more fragile than polymersomes, and their preparation is more delicate.

Lorenceau and Utada [37] used water in oil in water (W/O/W) double emulsions as templates to form monodisperse polymersomes by using a diblock copolymer poly(normal-butyl acrylate)-poly(acrylic acid) (PBA-PAA) in a capillary microfluidic device. The amphiphilic PBA-PAA in the middle fluid stabilized the two oil-water interfaces, and self-assembled on the interface during the dewetting oil phase upon solvent evaporation. The concentration of the amphiphilic molecules was a key control variable in the fabrication of polymersomes in this fabrication process [38]. If the concentration was lower than the amount required to fully cover the interfaces, the polymersomes were unstable. However, too much excess created a depletion interaction. 

**Figure 14:**
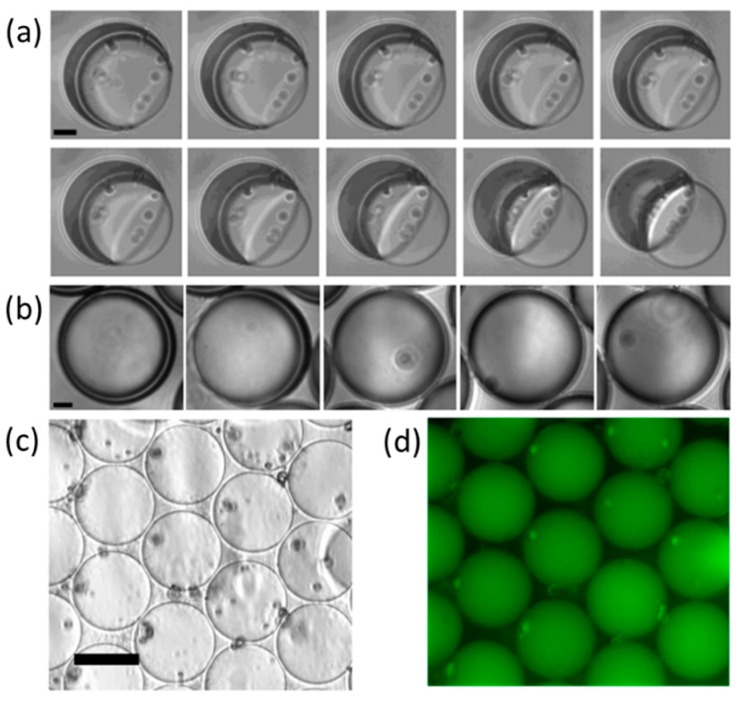
(**a**) Bright-field microscope images of a PEG(5000)-b-PLA(5000) polymersome undergoing dewetting transition; (**b**) Bright-field microscope images of a PEG(1000)-b-PLA(5000) polymersome undergoing the evaporation of the organic solvent shell; (**c**,**d**) Bright-field and fluorescence microscope images of a dried capsule formed from the PEG(1000)-b-PLA(5000) diblock copolymer. Pictures are from [114]. Reproduced with permission from Shum, H.C., et al., *Journal of the American Chemical Society*; published by American Chemical Society, 2008.

By integrating the advantages of the diblock copolymer and the microdroplet based-on microfluidic technology, the polymersome can be easily fabricated with excellent encapsulation efficiency, high levels of loading and tunable wall properties. For example, since the three fluids can be controlled individually, the encapsulate can contain the same amount of active material in each polymersomes and guarantee that the encapsulation efficiency will reach 100% [38]. In addition, the character of each block in the diblock copolymer can be tuned to fit the desired application. For example, tuning the molecular weight ratio of the hydrophilic and the hydrophobic blocks, the wetting angle of the polymer-containing solvent phase on the polyersomes will change in the emulsion-to-vesicles transition [114], as shown in Figure 14. The polymerization degree of the individual diblock molecules will affect the membrane thickness, whereas the elasticity and permeability of the membrane can be adjusted by changing the glass transition temperature of the hydrophobic block [145,146,147].

**Figure 15:**
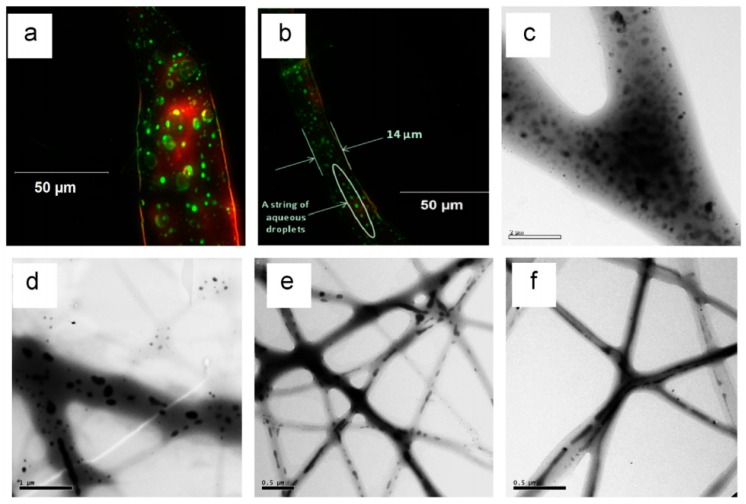
Morphological evaluation of the water phase in electrospun fibers collected at different locations of the emulsion jet path: (**a**,**b**) fluorescence microscopy images of fibers; (**c**–**f**) TEM micrographs at different magnifications. Pictures are from [148]. Reproduced with permission from Wang, C., et al., *Materials Letters*; published by Elsevier, 2014.

Core shell structured fibers are also excellent delivery vehicles for medicines. Wang et al. fabricated fibers with core shell structures by emulsion electrospinning [148]. The water in oil (W/O) emulsions were composed of deionized water or a phosphate buffer saline, and a poly(lactic-co-glycolic acid) (PLGA)/chloroform solution that contained a surfactant was used as a module to produce core shell structured fibers. This study demonstrated the evolution of the core shell structured fibers by investigating the water phase morphology in jets or fibers at different locations of the jet or fiber trajectory during the electrospinning process, as shown in Figure 15. They found that the water phase in emulsion jets experienced multi-level stretching and broke up. The Rayleigh capillary instability and the solvent evaporation rate significantly affected the breakup of water droplets. 

Since the mechanical properties of core shell microparticles made from materials with dramatically different elastic properties are also important factors in medical applications, the mechanical properties of these microparticles were measured and predicted by a microfluidic approach [149]. By forcing the particles through a tapered capillary and analyzing their deformation, the shear and compressive moduli were easily measured in one single experiment. The results showed that the moduli of these core shell structures were determined both by the material composition of the core shell microparticles and by their microstructures.

## 4. Conclusions

Droplet microfluidics can provide environments with properties of exceptional control and good stability, which can be used for performing microparticles synthesis with unique properties. We reviewed the different microfluidic systems for controlling droplet formation, the influencing factors for regulating droplet size and structure, and droplets solidification methods according to different materials. To make the applications of droplet microfluidics in preparation of micro- and nanoparticles more clear, we introduced the preparation methods of different materials, different structures and different functional microspheres in detail. Because these particles are characterized by particle size uniformity, structure controllability and component controllability, they are more widely applicable than those prepared by traditional methods, especially in new medicine (embolization treatment of tumor, drug controlled-release, multi-drug loading microspheres), adsorption separation, dual-functionalized optical, electrical and magnetic devices, etc. This technique has become a powerful platform in material fabrication, and biological and medical research. However, one obstacle in the practical application in microparticles preparation is their low-throughput, which limits the production efficiency. Recent advances have partially overcome this barrier by parallelizing droplet generation and enhanced the production rate by a factor of 100, but these parallel experiments were used primarily for the generation of spherical single droplets and didnot report on the generation of non-spherical, double or multi-emulsion, and nor did perform on droplets in situ curing parallelization. More efforts are needed to resolve these technical issues. Given the distinctive properties of this technique and its tremendous demand in applications, droplet microfluidics will fundamentally modify the future of micro- and nano-manufacturing and drug delivery.
